# Snake Tooth in the Finger!

**DOI:** 10.5005/jp-journals-10071-23116

**Published:** 2019-01

**Authors:** B Sadananda Naik

**Affiliations:** Alva's Health Centre, Moodabidri, Karnataka, India

**Keywords:** Envenomation, Non-venomous snakes, Retained snake tooth

## Abstract

**How to cite this article:** Naik BS. Snake tooth in the finger! Indian Journal of Critical Care Medicine, January 2019;23(1): 58.

A 60-year-old lady was referred by a tertiary care hospital for follow up. This lady had been treated for severe viper envenomation with several complications like AKI, hepatic derangement, and coagulopathy. She was discharged home after full recovery. On examination, there was a small bite wound in her finger which looked clean and healthy. I congratulated her for not getting any residual tissue or organ damage which usually follows a severe snake envenomation. But, she hardly gave any response; kept gazing at something, looked very depressed. She was unusually silent, but I could perceive an untold fear in her eyes.

This patient was quite well until a group of relatives visited her a few days back. Since their visit, she looked very depressed, avoided any conversations with family members, hardly took any food and kept looking at her finger all the time. Suddenly, I remembered a snake bite patient telling me about the so-called “retained snake tooth in the bite wound”. I instantaneously understood the mystery surrounding this patient and with all the confidence in the world. I told the patient loudly that “ there is no snake tooth retained in your finger”. Suddenly, the lady got up from her wheelchair with all the smile in her face and the tears trickling down her eyes. I consoled her and convinced her that, it is the snake venom which is lethal and there is no snake tooth retained in her finger.

On persistent inquiry, the patient told me the story which bugged her mind. One of her relative who visited told that the viper snakes do leave the tooth at the bite site while biting and this retained tooth leads to decaying of the entire limb gradually and surely. He also told that the practitioners of modern medicine are quite ignorant about this fact and only a few snake charmers know the art of removing the retained tooth. Incidentally, there was an ecchymotic patch on her arm on the same side which cemented her fear.

Few non-poisonous snakes like python and very rarely vipers could leave their teeth while biting which may result in local sepsis.^1-3^ Banking on this rare occurrence, the snake charmers exploit the patients. In India, the treatment of venomous snake bite is marred by several limitations, especially at the rural areas. Though there has been a sea of changes in this regard, the myths surrounding the snake bite and envenomation still remains a big challenge. As narrated in this incidence, even the well-educated people also fall prey to these myths and beliefs.
